# Comprehensive Evaluation on Teachers' Knowledge Sharing Behavior Based on the Improved TOPSIS Method

**DOI:** 10.1155/2022/2563210

**Published:** 2022-04-21

**Authors:** Xiaojuan Yu, Dianshun Hu, Na Li, Yan Xiao

**Affiliations:** ^1^School of Mathematics and Statistics, Central China Normal University, Wuhan 430079, China; ^2^College of Mechanical Engineering, Chongqing University of Technology, Chongqing 400054, China; ^3^School of Business, Macau University of Science and Technology, Macau 999078, China

## Abstract

Knowledge sharing among teachers is one of the important ways to improve their teaching and research ability. From the perspectives of teachers' knowledge sharing willingness, knowledge sharing ability, knowledge sharing environment, knowledge sharing technology support, knowledge sharing effect, and so forth, this paper constructs a teacher knowledge sharing behavior evaluation model, develops a knowledge sharing behavior evaluation index system, and proposes a comprehensive teacher's knowledge sharing behavior evaluation method based on the improved entropy-TOPSIS method. This is a comprehensive evaluation method combining subjective and objective weights, which avoids the subjectivity of traditional expert evaluation methods and other multilevel and multi-index weight determination methods and makes the evaluation results more objective, accurate, and more realistic. Finally, by taking the example of evaluating knowledge sharing behavior of teachers in a university of Chongqing, China, this paper verifies the feasibility and practicability of the proposed comprehensive teachers' knowledge sharing behavior evaluation system and method.

## 1. Introduction

With the advent of the knowledge economy era, knowledge resources and knowledge management ability have become an important source of core competitiveness of organizations and individuals [1, 2]. As practitioners in education, teachers are innovators and disseminators of professional knowledge, accumulating and creating knowledge in specific educational and teaching contexts through “cognition in action” and “reflection in action,” which play important roles in the development of knowledge economy. With the continuous differentiation and depth of knowledge, teachers also need to constantly learn and supplement new knowledge to guarantee the competitiveness and value of their knowledge resources. Teachers' knowledge sharing not only helps to excavate and reveal teachers' individual knowledge and improve teachers' individual ability but also helps to realize the transformation of teachers' individual knowledge into group public knowledge and further enrich the practical knowledge base of the teacher community, thus providing important knowledge resources for the development of school organizations, which is of great significance to individual teachers, teacher groups, and school organizations [3, 4].

With the in-depth development of specialized subject, knowledge increasingly embodies the characteristics of complexity, intersection, and synthesis. Meanwhile, there are large knowledge gaps among teachers due to differences in educational background, knowledge stock, learning ability, thinking patterns, academic expertise, and personality traits [5]. Knowledge sharing among teachers is an important way to help teachers quickly understand, comprehend, and master knowledge, and, whether as individuals or as teacher community, its essence is the process of teachers transferring, communicating, and sharing knowledge [6]. However, compared with the attention paid to teachers' knowledge sharing, teachers' knowledge sharing behavior has encountered dilemmas such as absence, low end, and ineffectiveness in real practice. Teachers are constrained by factors such as egoism and altruism, subjective sharing desire and objective conditions, self-worth of knowledge, and lack of resource facilitation, which hinder university teachers' willingness and action to share knowledge, thus leading to a lack of effective knowledge exchange and cooperation [7, 8]. Therefore, stimulating internal drive of teachers to participate in knowledge sharing and mobilizing their willingness to participate in knowledge sharing behavior can promote the effective realization of teachers' knowledge sharing, thus promoting their personal development and the improvement of school knowledge management capability. Among them, how to effectively and accurately evaluate teachers' knowledge sharing behavior is the key link to improve the level of knowledge sharing among teachers. While exploring various strategies to improve knowledge level of teachers, schools must first understand the development status of teachers' knowledge sharing behavior and take the current situation of teachers' knowledge sharing behavior as the focus of improving teachers' knowledge level, which requires clarifying the methods for monitoring and evaluating the behavior of teachers' knowledge sharing. Therefore, this paper focuses on the issue of evaluation of teachers' knowledge sharing behavior to clarify the current situation of teachers' professional knowledge, so as to improve the quality of teachers' development.

The results of literature retrieval show that although there are some achievements on knowledge sharing behavior evaluation, teachers' knowledge management performance evaluation, and teachers' knowledge sharing influencing factors, there are still few achievements of in-depth research on tacit knowledge sharing behavior evaluation of teachers. In addition, current research on teachers' knowledge sharing behavior is dominated by theoretical studies and less by quantitative empirical studies. Accordingly, based on the systematic study of the influencing factors and evaluation indicators of teachers' knowledge sharing behavior, this paper constructs an evaluation model of knowledge sharing behavior and implements empirical analysis using the entropy weight method and the TOPSIS method. This work is beneficial for managers to provide effective incentives and management measures for teachers' knowledge sharing behavior in the school, which can provide theoretical guidance for improving teachers' knowledge sharing ability.

The structure of this paper is organized as follows: Section 2 reviews the related works about evaluation of teachers' knowledge sharing behavior. Then, the indicator system of teachers' knowledge sharing behavior evaluation is developed in Section 3. In Section 4, the evaluation model of teachers' knowledge sharing behavior is proposed.  Section 5 presents a real case to show the feasibility and effectiveness of the proposed evaluation method. Finally, Section 6 presents the conclusion and future work.

## 2. Literature Review

The issue of knowledge management in education has received increasing attention from the society and academia, and the researches related to teachers' knowledge sharing have become a research hotspot. In terms of knowledge sharing behavior and its influencing mechanisms, Cummings and Teng [9] argued through empirical studies that the degree to which knowledge can be expressed through words or graphics determines the difficulty and ease of knowledge sharing; that is, the easier it is to express, the more favorable it is to share. Hu and Liu [10] used the complete information game approach to find the subgame perfect Nash equilibrium solution for the game between each participant in the knowledge alliance. Tseng and Kuo [8] found that the knowledge sharing behavior of teacher groups in online communities was significantly influenced by social relationships and community affiliation management. Liu et al. [11] established a single-group and multigroup dynamic game model of knowledge sharing and analyzed the evolutionary stabilization strategy of this model. Caskova and Chudy [12] explored the effects of the school culture on teachers' career at the beginning of their teaching and pedagogical knowledge sharing. Wu et al. [13] established the sharing behavior model and shared utility function, based on which experimental simulations of the model were conducted to summarize and analyze the knowledge sharing and motivation rules of organizational individuals. Mollazehi and Karimi [14] identified and validated the factors affecting teacher's knowledge sharing through information and communication technology (ICT).

For the study on teachers' knowledge sharing behavior evaluation, Sun et al. [15] clarified the connotation and composition of tacit knowledge sharing ability of university teachers based on two levels of process and elements and thus constructed an index system for tacit knowledge sharing ability evaluation of university teachers. Zhang et al. [16] conducted a specific study on the knowledge sharing ability of teacher scientific research teams from a holistic network perspective, including network stickiness measurement, centrality measurement, core-edge measurement, cohesive subgroups, and structural hole measurement. McChesney and Aldridge [17] described the development and validation of a new instrument to assess teachers' perceptions of the impact of professional knowledge sharing. Zhao and Wang [18] constructed a tacit knowledge sharing model for university teachers based on their knowledge sharing intention, sharing ability, sharing atmosphere, and sharing effect and then used AHP method to measure and analyze the weights of the index system. Asghar and Naveed [19] evaluated the psychometric properties of the Knowledge Sharing Behavior Scale (KSBS) in an academic context, and the results indicated that KSBS is not a valid instrument for measuring knowledge sharing behavior within an academic environment. Li and Qin [20] provided a model framework for teachers to promote students' entrepreneurial motivation through knowledge sharing. Kularajasingam and Subramaniam [21] stated that university academics' knowledge sharing behavior and social intelligence are significant in improving their performance through their grasp of competencies, and they applied a mediation model among university teachers to investigate the impact of knowledge sharing behavior and social intelligence of university academics on their performance. Wang et al. [22] examined factors explaining rural teachers' sharing behavior regarding digital educational resources, both within and outside school, as posited by combining motivation theory and the integrative model of behavior prediction.

The above research provides a very valuable reference for the evaluation of teachers' knowledge sharing behavior. However, due to the professional and tacit characteristics of teachers' knowledge, the evaluation of teachers' knowledge sharing behavior has multiobjective attribute, and it is usually difficult to balance between different goals; that is, it is difficult to have one evaluation scheme whose indicators are better than others [15]. Therefore, the evaluation of teachers' knowledge sharing ability needs a quantitative comprehensive evaluation method to improve the validity of the evaluation process and reduce subjectivity. Thus, this paper constructs an evaluation indicator system of teachers' knowledge sharing behavior and proposes a comprehensive evaluation method based on entropy weight method and TOPSIS method and then verifies the feasibility and effectiveness of this evaluation system and evaluation method through practical cases.

## 3. The Indicator System of Teachers' Knowledge Sharing Behavior Evaluation

The evaluation system of teachers' knowledge sharing efficiency is a complex evaluation system, and it is unrealistic to describe the essence and rule of the whole process of teachers' knowledge sharing to control it to reach the predetermined goals of individual teachers and school organizations, which requires the establishment of a massive index system [18]. One index can only reflect a certain attribute of the knowledge sharing behavior evaluation system. Therefore, a reasonable evaluation indicator system can only be formed by selecting the main indicators in a reasonable way to comprehensively evaluate the knowledge sharing behavior of teachers within a reasonable cost range.

Although the performance of knowledge sharing behavior is related to the contribution of individual teacher, knowledge sharing among individual teachers is more influenced by some difficult-to-quantify factors of interactions among members, and the results of knowledge sharing are not immediately apparent, showing a certain lag phenomenon [23, 24]. When evaluating knowledge sharing behavior, it is necessary to consider the impact of difficult-to-quantify interrelationships among teachers and their roles in the overall knowledge sharing process. Therefore, for teachers' knowledge sharing, outcome-based indicators can only reflect part of the final results of knowledge sharing behavior but less consider the process. In fact, the more important aspect of knowledge sharing behavior is the behavioral activities and the interactive coordination between individuals, which is extremely crucial. In addition, from the practice of teachers' knowledge sharing behavior, the cognitive gap between individual teachers and the knowledge sharing environment are objective factors that affect knowledge sharing behavior; thus the evaluation of teachers' knowledge sharing behavior should consider the above factors [25, 26].

Based on the above considerations, this paper comprehensively evaluates teachers' knowledge sharing behavior from five dimensions, namely, knowledge sharing willingness, knowledge sharing ability, knowledge sharing environment, knowledge sharing technology support, and knowledge sharing effect. The logical process of this index system is as follows: First, before the beginning of knowledge sharing, teachers' knowledge sharing willingness is the determining factor for their decision to implement knowledge sharing behavior. Second, in the process of knowledge sharing, teachers' individual knowledge sharing ability, external knowledge sharing environment, and knowledge sharing technology support are the key subjective and objective factors affecting knowledge sharing behavior. Third, all these subjective and objective factors are ultimately reflected in the effect of knowledge sharing behavior. When the knowledge sharing has produced certain effects, the knowledge sharing behavior of teachers can be reflected in its entirety. Further, through analyzing the characteristics and connotations of the five dimensions and following the principles of systemic, scientific, operability, and comparability of the evaluation indicator system, the evaluation indicator system of teachers' knowledge sharing behavior is constructed, as shown in Table 1.

## 4. Evaluation Model of Teachers' Knowledge Sharing Behavior

Teachers' knowledge sharing behavior is a complex systematic process, and the whole process involves various theories such as transaction cost theory, cognitive psychology, and organizational behavior [27]. Teachers' knowledge sharing behavior evaluation should realize the promoting function of the evaluation process and results on the knowledge exchange behavior among teachers and then improve teachers' individual knowledge ability and school knowledge management level through knowledge sharing behavior evaluation, which shows that teachers' knowledge sharing behavior evaluation is a multiobjective evaluation problem. Many multiobjective evaluation methods have been proposed [28, 29], and when teachers' knowledge sharing is used as the evaluation object, the evaluation conclusions obtained by various methods often differ largely since the rubric used is often subjective and ambiguous, which undoubtedly brings great difficulties to the evaluation of teachers' knowledge sharing behavior.

After the evaluation indicator system of teachers' knowledge sharing behavior is determined, its evaluation results mainly depend on two factors: one is the determination of evaluation index weights, and the other is the choice of comprehensive evaluation methods. Traditional knowledge sharing behavior evaluation methods often rely on the subjective judgments of organizers and experts to determine the weights of each index, and due to the great differences in professional personal experience and mastering information, which are subjective, the weight scores given by different individuals for the same evaluation index often differ greatly, resulting in great distortions of evaluation results and even wrong decisions [18]. Based on the above considerations, this paper combines the subjective judgment of experts with the objective situation of teachers' knowledge sharing behavior, determines the weights of the evaluation indexes of enterprise culture implementation by entropy weight method, and adjusts the subjective deviation of expert judgment by scientific weight coefficients and then adopts the improved ideal point (TOPSIS) as the comprehensive evaluation method to derive the final ideal evaluation results.

### 4.1. Entropy Weight Method and TOPSIS Method

The concept of entropy originated from thermodynamics and was later introduced into information theory by Shannon. According to the definition and principle of entropy, the entropy value can be used as a measure of the amount of effective information provided by the system, representing the degree of disorder of the system. Entropy weight method is an evaluation method combining qualitative and quantitative analysis, which is an objective weighting method [30, 31]. The entropy weight method determines the indicator weights according to the amount of information conveyed to the decision-maker by each index. For an index, the entropy value can be used to judge the degree of dispersion of an index. If an index's information entropy value is smaller, the dispersion degree of the index is greater, and the influence of the index on the comprehensive evaluation is greater. If the values of an index are all equal, the index does not work in the comprehensive evaluation. For the evaluation problem, suppose that there are *m* evaluation objects and *n* evaluation indicators, and the initial evaluation matrix R=(*r*_*ij*_)_*m*×*n*_ is obtained, and *r*_*ij*_ denotes the value of the *j* evaluation indicator of the *i* evaluation object; then the entropy value *e*_*j*_ of an indicator *r*_*j*_ is

(1) $$\begin{array}{c} e_{j}=-\frac{1}{\ln\;m}\sum_{i=1}^{m}\delta_{ij}\ln\;\delta_{ij}, \end{array}$$

where *δ*_*ij*_=*r*_*ij*_/∑_*i*=1_^*m*^*r*_*ij*_ and *δ*_*ij*_ denotes the proportion of the *i* participant to the indicator under the *j* indicator. According to the definition and principle of entropy weight method, when the entropy value of an index is larger, it means that the less effective information provided by the index, the smaller the role in the comprehensive evaluation, and its weight is smaller; conversely, the larger the entropy value, the more effective information provided by the index and the larger the role in the comprehensive evaluation, and its weight is larger [31]. Therefore, it is scientific and credible to use the entropy weight method to assign index weight during the analysis of multiple index evaluation problems.

TOPSIS method is a common method in system engineering, mainly for multiobjective evaluation and decision analysis of finite solutions, and it has many advantages, such as no requirement for sample content, no requirement for sample data distribution, simple calculation process, and intuitive and easy analysis of results, which makes TOPSIS method widely and effectively used in many fields, such as economy, management, and engineering technology. [28, 32, 33]. For the evaluation of teachers' knowledge sharing behavior, the TOPSIS method has no strict restrictions on data distribution and sample content indicators. It is suitable for small sample data and large-scale system data with multiple evaluation units and multiple indicators, which has the advantage of being real, intuitive, and reliable. The core idea and procedure of TOPSIS are as follows: Firstly, the initial data is dimensionless processed to make the data objectively and truly reflect the gap between the evaluation objects; then, the positive and negative ideal values are determined according to the size of the standardized data of each index; next, the weighted Euclidean Distance between the evaluation objects and the positive and negative ideal values is calculated, from which the closeness of each evaluation object to the best state is derived; finally, the ranking of each evaluation object is measured. In this paper, when applying TOPSIS method for comprehensive evaluation, to address the problem of cumbersome calculation of Euclidean Distance from evaluation objects to the positive and negative ideal point, the decision matrix of TOPSIS method is normalized to simplify the calculation of the positive and negative ideal solution; meanwhile, the concept of relative closeness between the index value and ideal solution of each evaluation object is introduced, and each evaluation object is ranked according to the size of its relative closeness.

### 4.2. Comprehensive Evaluation Process of Entropy Weight-TOPSIS Method

The evaluation steps of the improved entropy weight-TOPSIS method are as follows:

For the evaluation problem with *m* teachers to be evaluated and *n* evaluation indicators, there is the initial data matrix R=(*r*_*ij*_)_*m*×*n*_.

(1) The normative matrix Q=(*q*_*ij*_)_*m*×*n*_ is obtained by dimensionless treatment of the R matrix; that is,
  (2) $$\begin{array}{c} q_{ij}=\frac{r_{ij}}{\sqrt{\sum_{i=1}^{m}r_{ij}{}^{2}}},\;i=1\text{,}2\text{,}3...\text{,}m,\;j=1\text{,}2\text{,}3...\text{,}n. \end{array}$$
(2) Calculate *δ*_*ij*_, which is the weight of the *j* indicator of the *i* participant.
  (3) $$\begin{array}{c} \delta_{ij}=\frac{q_{ij}}{\sum_{i=1}^{m}q_{ij}}. \end{array}$$
(3) Calculate the entropy value *e*_*j*_ of the *j* indicator.
  (4) $$\begin{array}{c} e_{j}=-\frac{1}{\ln\;m}\sum_{i=1}^{m}\delta_{ij}\ln\;\delta_{ij}\text{,}\;\left(j=1\text{,}2\text{,}3...\text{,}n\right)\text{,} \end{array}$$
where 0 ≤ *e*_*j*_ ≤ 1.
(4) Calculate the difference coefficient *e*_*j*_ for the *j* indicator.
  (5) $$\begin{array}{c} {\text{g}}_{j}=1-e_{j}. \end{array}$$
For the *j* indicator, the larger the difference coefficient *χ*_*j*_, the greater the role of the indicator in the program evaluation; conversely, the smaller *χ*_*j*_ is, the smaller the role of the indicator in the program evaluation is.
(5) Calculate the weight *w*_*j*_ of the *j* indicator.
  (6) $$\begin{array}{c} w_{j}=\frac{{\text{g}}_{j}}{\sum_{j=1}^{n}{\text{g}}_{j}}. \end{array}$$
(6) Construct the weighted data matrix Z=(*z*_*ij*_)_*m*×*n*_, where element *z*_*ij*_ is defined as follows:
  (7) $$\begin{array}{c} z_{ij}=w_{j}q_{ij}. \end{array}$$
(7) Determine the positive ideal value *R*^+^ and the negative ideal value *R*^−^ of the index. The traditional TOPSIS method of the positive and negative ideals is more complex due to the values, which makes it difficult to calculate the Euclidean Distance of each evaluation scheme to the positive and negative ideals. For this reason, without affecting the evaluation problem to derive the final evaluation results, this paper makes improvements to the TOPSIS method to simplify the calculation. The value of *q*_*ij*_ in the normative matrix Q is taken as [0,1]. Here we specify that the highest preferred target attribute value *q*_*ij*_=1 and the lowest preferred target attribute value *q*_*ij*_=0. Thus, it can be known that *z*_*j*_^*∗*^=*w*_*j*_ and *z*_*j*_^−^=0; then the positive and negative ideal solutions are as follows:
  (8) $$\begin{array}{c} {\text{Z}}^{-}=\left(z_{1}{}^{-}\text{,}z_{2}{}^{-}...\text{,}z_{j}{}^{-}\right)=\left(0\text{,}0...\text{,}0\right)\text{,}\\ {\text{Z}}^{*}=\left(z_{1}{}^{*}\text{,}z_{2}{}^{*}...\text{,}z_{j}{}^{*}\right)=\left(w_{1}\text{,}w_{2}...\text{,}w_{j}\right)\text{.} \end{array}$$
(8) Calculate the Euclidean Distance of each evaluation object to the positive and negative ideal points, and the distance formula uses Euclidean formula.
  (9) $$\begin{array}{c} D_{i}{}^{-}=\sqrt{\sum_{j=1}^{n}{\left(z_{ij}-z_{j}{}^{-}\right)}^{2}}=\sqrt{\sum_{j=1}^{n}w_{ij}{}^{2}{\left(q_{ij}-0\right)}^{2}},\\ D_{i}{}^{*}=\sqrt{\sum_{j=1}^{n}{\left(z_{ij}-z_{j}{}^{*}\right)}^{2}}=\sqrt{\sum_{j=1}^{n}w_{ij}{}^{2}{\left(q_{ij}-1\right)}^{2}},\;i=1\text{,}2\text{,}3...\text{,}m,\;j=1\text{,}2\text{,}3...\text{,}n. \end{array}$$
(9) Calculate the relative closeness between the index value and the ideal solution of each evaluation object *ψ*_*i*_.
  (10) $$\begin{array}{c} \psi_{i}=\frac{D_{i}{}^{-}}{D_{i}{}^{*}+D_{i}{}^{-}}. \end{array}$$
(10) The schemes are ranked by the magnitude of relative closeness, and the greater the relative closeness *ψ*_*i*_, the higher the level of knowledge sharing of the evaluated teachers; the greater *ψ*_*i*_ is, the lower the level of knowledge sharing of the evaluated teachers is.

## 5. Case Study

This paper selects a well-known university in Chongqing, China, as an example of teacher knowledge sharing evaluation work and illustrates the evaluation process of the teacher knowledge sharing evaluation model and method. This university is a multidisciplinary university with distinct engineering characteristics, and it focuses on cultivating application-oriented compound high-quality professionals with innovative spirit and practical ability. This university has more than 2300 teachers and staffs, and it strongly focuses on and supports knowledge sharing behaviors among teachers to improve individual teachers and the university's overall knowledge management. In the process of evaluating the knowledge sharing behavior of teachers in this university, the above evaluation system is used to comprehensively evaluate the knowledge sharing level of four teachers, A, B, C, and *D*, and then give the ranking of each teacher's knowledge sharing behavior.

In this paper, an expert panel is formed by several experts in higher education systems and knowledge management, and the expert panel scores each teacher individually according to the evaluation indicator system proposed in this paper. The range of scores is 1 − 5, where higher scores indicate a higher level of knowledge sharing behavior of a teacher on an indicator. The final combined scores of the expert are obtained in Table 2.

1. Since the scores in the expert rating scale are all dimensionless data, there is no need for dimensionless processing here.
2. According to equation (3), the weight *δ*_*ij*_ of the *i* participating teacher in the *j* indicator is calculated to obtain Table 3.
3. According to equations (4)–(6), the entropy value, variation coefficient, and weight of each evaluation index are obtained, and the results are shown in Table 4.
4. As shown in Table 5, the weighted normalized data matrix is obtained from equation (7).
5. As shown in Table 6, the relative closeness and its ranking table can be obtained from equations (8)–(10).

The ranking result of the relative closeness *c*_*i*_ shows that the four university teachers' knowledge sharing behavior level is B〉D〉A〉C in turn, which is consistent with the intuitive judgment of experts and school colleagues about the four teachers. Meanwhile, the results are consistent with those of the unsimplified traditional TOPSIS method, so the evaluation process of the improved TOPSIS method is more concise and efficient, and the evaluation results have higher credibility and practicality.

## 6. Conclusions

Knowledge sharing behavior evaluation can help schools and teachers to understand their knowledge sharing levels and thus provide decision-making input for better policy formulation, environment optimization, organization construction, and incentive behavior. Teachers' knowledge sharing behavior evaluation has become a hot focus of attention in education and academia at home and abroad, yet there is little research on the quantitative evaluation of teachers' knowledge sharing behavior. From a systematic perspective of combining process and result, this paper constructs an evaluation indicator system of teachers' knowledge sharing behavior containing five dimensions of knowledge sharing willingness, knowledge sharing ability, knowledge sharing environment, knowledge sharing technology support, and knowledge sharing effect, which is proved in practice to be able to evaluate teachers' knowledge sharing level scientifically, systematically, and comprehensively. The entropy weight method is used to revise experts' experience scoring results and determine the weights of knowledge sharing behavior evaluation index, which avoids the subjectivity of the traditional expert evaluation method and other multilevel and multi-index weight determination methods and makes the evaluation results more objective, accurate, and more in line with reality. Compared with the traditional TOPSIS method, the improved TOPSIS method has a more streamlined, efficient, and well-organized calculation process. In summary, the evaluation system and evaluation method of teachers' knowledge sharing behavior established in this paper are reasonable and practical, which can not only judge the comprehensive level of teachers' knowledge sharing ability but also analyze the strengths and weaknesses factors of each teacher's knowledge sharing, to provide a basis for decision-making to improve teachers' knowledge sharing level. Of course, the empirical research in this paper still has its limitations, such as insufficient sample sources and insufficient sample size. In the future work, the empirical analysis is needed to further verify the advantages of the evaluation model and method in this paper.

## Figures and Tables

**Table 1:** The evaluation indicator system of teachers' knowledge sharing behavior.

Evaluation objects	Level 1 indicators	Level 2 indicators
Teachers' knowledge sharing behavior evaluation	Knowledge sharing willingness	Self-efficiency u11
		Interpersonal relationships u12
		Gaining respect u13
		Knowledge sharing recognition u14
		Position in the organization u15
	Knowledge sharing ability	Knowledge recognition ability u21
		Knowledge transfer ability u22
		Knowledge receiving ability u23
		Knowledge innovation ability u24
	Knowledge sharing environment	Knowledge exchange atmosphere u31
		Knowledge sharing incentive policy u32
		Knowledge sharing culture u33
		Trust among teachers u34
		Intellectual property policy u35
	Knowledge sharing technology support	Knowledge sharing platform u41
		Knowledge exchange medium u42
		Knowledge sharing map u43
		Knowledge base u44
	Knowledge sharing effect	Gaining valuable knowledge u51
		Knowledge sharing satisfaction u52
		Personal capability enhancement u53
		Colleague's approval u54
		Organizational performance improvement u55

**Table 2:** Original data of expert panel scoring.

	u11	u12	u13	u14	u15	u21	u22	u23	u24	u31	u32	u33	u34	u35	u41	u42	u43	u44	u51	u52	u53	u54	u55
A	3	3	4	2	3	3	4	3	2	4	3	3	2	3	4	3	2	2	2	3	2	3	2
B	3	3	3	3	3	3	3	4	4	3	4	3	2	4	3	4	3	3	3	4	2	3	3
C	5	4	3	3	4	3	3	4	3	3	4	2	2	3	2	3	1	2	1	2	2	2	2
D	3	4	3	3	3	4	3	4	3	3	3	3	3	2	3	3	3	2	2	3	2	3	3

**Table 3:** The weight of the *i* participating teacher in the *j* indicator.

	u43	u44	u51	u52	u53	u54	u55	u43	u44	u51	u52	u53	u54	u55	u43	u44	u51	u52
A	0.214	0.214	0.307	0.181	0.231	0.231	0.307	0.202	0.168	0.307	……	0.250	0.249	0.250	0.273	0.249	0.231	0.200
B	0.214	0.214	0.231	0.273	0.231	0.231	0.231	0.266	0.334	0.231	……	0.375	0.334	0.250	0.273	0.334	0.231	0.300
C	0.358	0.286	0.231	0.273	0.307	0.231	0.231	0.266	0.249	0.231	……	0.125	0.168	0.250	0.181	0.168	0.231	0.200
D	0.214	0.286	0.231	0.273	0.231	0.307	0.231	0266	0.249	0.231	……	0.250	0.249	0.250	0.273	0.249	0.307	0.300

**Table 4:** Entropy values, variation coefficients, and weights of each evaluation index.

	u43	u44	u51	u52	u53	u54	u55	u43	u44	u51	u52	u53	u54	u55	u43	u44	u51	u52
Ej	0.979	0.992	0.994	0.99	0.994	0.995	0.98	0.994	0.992	0.99	……	0.953	0.98	1	0.99	0.98	0.994	0.985
Gj	0.021	0.008	0.006	0.01	0.006	0.005	0.02	0.006	0.008	0.01	……	0.047	0.02	0	0.01	0.02	0.006	0.015
Wj	0.057	0.022	0.016	0.027	0.016	0.014	0.055	0.016	0.022	0.027	……	0.128	0.055	0	0.027	0.055	0.016	0.041

**Table 5:** Weighted normalized data matrix.

	u43	u44	u51	u52	u53	u54	u55	u43	u44	u51	u52	u53	u54	u55	u43	u44	u51	u52
A	0.172	0.065	0.065	0.055	0.049	0.0409	0.219	0.049	0.044	0.109	……	0.257	0.164	0	0.082	0.164	0.049	0.082
B	0.172	0.065	0.049	0.082	0.049	0.041	0.164	0.065	0.087	0.082	……	0.385	0.219	0	0.082	0.219	0.049	0.123
C	0.286	0.087	0.049	0.082	0.066	0.041	0.164	0.065	0.065	0.082	……	0.128	0.1099	0	0.055	0.109	0.049	0.082
D	0.172	0.087	0.049	0.082	0.049	0.055	0.164	0.065	0.065	0.082	……	0.257	0.164	0	0.082	0.164	0.065	0.123

**Table 6:** Relative closeness *ψ*_*i*_ and ranking results table.

Evaluation object	A	B	C	D
*ψ*_*i*_	0.178566	0.323158	0.119644	0.213466
Ranking results	3	1	4	2

## Data Availability

The data used to support the findings of this study are included within the article.

## References

[1] Sun Y., Liu J., Ding Y. (2020). Analysis of the relationship between open innovation, knowledge management capability and dual innovation. *Technology Analysis & Strategic Management*.

[2] Jiafu S., Yu Y., Tao Y. (2018). Measuring knowledge diffusion efficiency in R&D networks. *Knowledge Management Research and Practice*.

[3] Kaya T., Erkut B. (2018). The tacit knowledge capacity of lecturers: a cross-country comparison. *Electronic Journal of Knowledge Management*.

[4] Gu X., O’Connor J. (2019). Teaching ‘tacit knowledge’in cultural and creative industries to international students. *Arts and Humanities in Higher Education*.

[5] Yu D., Zhou R. (2015). Tacit knowledge sharing modes of university teachers from the perspectives of psychological risk and value. *International Journal of Higher Education*.

[6] Verloop N., Van Driel J., Meijer P. (2001). Teacher knowledge and the knowledge base of teaching. *International Journal of Educational Research*.

[7] Edge K. (2013). Rethinking knowledge management: strategies for enhancing district-level teacher and leader tacit knowledge sharing. *Leadership and Policy in Schools*.

[8] Tseng F.-C., Kuo F.-Y. (2014). A study of social participation and knowledge sharing in the teachers’ online professional community of practice. *Computers & Education*.

[9] Cummings J. L., Teng B. S. (2003). Transferring R&D knowledge: the key factors affecting knowledge transfer success. *Journal of Engineering and Technology Management*.

[10] Hu Y., Liu X. (2009). Game analysis of knowledge sharing in knowledge alliance. *Science & Technology Progress and Policy*.

[11] Liu C., Shan W., Yu J. (2014). Type and evolutionarygame model of knowledge sharing within organizations. *Science Research Management*.

[12] Caskova K., Chudy S. (2021). Influence of school culture on pedagogical knowledge sharing between an education student and a training teacher. *SN Social Sciences*.

[13] Wu J. Z., Song S., Zhi F., Guang M. A. (2015). Games analysis and simulation in knowledge sharing based on knowledge contribution assessment and utility among organizational employees. *Journal of Industrial Engineering and Engineering Management*.

[14] Mollazehi A., Karimi F. (2019). Identification and validating factors affecting teachers’ knowledge sharing through ICT. *Sciences and Techniques of Information Management*.

[15] Sun D., Li Y., Yu D. (2015). Ability evaluation model of university teachers’ tacit knowledge sharing. *Information Science*.

[16] Zhang W., Zhang Q., Shan W. (2012). Research on measurement of knowledge sharing capabilities of university research team based on overall network perspective. *Science of Science and Management of S.& T.*.

[17] McChesney K., Aldridge J. M. (2019). A review of practitioner-led evaluation of teacher professional development. *Professional Development in Education*.

[18] Zhao L., Wang C. (2014). A research on university teachers’ tacit knowledge sharing model and evaluation index system. *China Educational Technology*.

[19] Asghar A., Naveed M. A. (2021). Psychometric Evaluation of Knowledge Sharing Behavior Scale in Academic Environment. *Library Philosophy and Practice*.

[20] Li J., Qin J. (2022). Effect of teachers’ knowledge sharing behavior on students’ entrepreneurial motivation in social media environment. *International Journal of Emerging Technologies in Learning (iJET)*.

[21] Kularajasingam J., Subramaniam A., Sarjit Singh D. K., Sambasivan M. (2022). The impact of knowledge sharing behaviour and social intelligence of university academics on their performance: the mediating role of competencies. *The Journal of Education for Business*.

[22] Wang J., Tigelaar D. E. H., Admiraal W. (2021). Rural teachers’ sharing of digital educational resources: from motivation to behavior. *Computers & Education*.

[23] Trusson C. R., Doherty N. F., Hislop D. (2014). Knowledge sharing using IT service management tools: conflicting discourses and incompatible practices. *Information Systems Journal*.

[24] Erickson G. S., Rothberg H., Carr C. (2003). Knowledge-sharing in value-chain networks: certifying collaborators for effective protection process. *Advances in Competitiveness Research*.

[25] Hew K. F., Hara N. (2007). Knowledge sharing in online environments: a qualitative case study. *Journal of the American Society for Information Science and Technology*.

[26] Booth S. E. (2012). Cultivating knowledge sharing and trust in online communities for educators. *Journal of Educational Computing Research*.

[27] Charband Y., Jafari Navimipour N. (2016). Online knowledge sharing mechanisms: a systematic review of the state of the art literature and recommendations for future research. *Information Systems Frontiers*.

[28] Zhang X., Su J. (2019). A combined fuzzy DEMATEL and TOPSIS approach for estimating participants in knowledge-intensive crowdsourcing. *Computers & Industrial Engineering*.

[29] Xiao Y., Li C., Song L., Yang J., Su J. (2021). A multidimensional information fusion-based matching decision method for manufacturing service resource. *IEEE Access*.

[30] Zhu Y., Tian D., Yan F. (2020). Effectiveness of entropy weight method in decision-making. *Mathematical Problems in Engineering*.

[31] He Y., Guo H., Jin M., Ren P. (2016). A linguistic entropy weight method and its application in linguistic multi-attribute group decision making. *Nonlinear Dynamics*.

[32] Jahanshahloo G. R., Lotfi F. H., Izadikhah M. (2006). Extension of the TOPSIS method for decision-making problems with fuzzy data. *Applied Mathematics and Computation*.

[33] Zhang X., Su J. (2018). An integrated qfd and 2-tuple linguistic method for solution selection in crowdsourcing contests for innovative tasks. *Journal of Intelligent and Fuzzy Systems*.

